# Association of Resilience and Social Networks with Pain Outcomes Among Older Adults

**DOI:** 10.1089/pop.2018.0199

**Published:** 2019-12-02

**Authors:** Shirley Musich, Shaohung S. Wang, Luke Slindee, Sandra Kraemer, Charlotte S. Yeh

**Affiliations:** ^1^ Research for Aging Populations, Optum, Ann Arbor, Michigan.; ^2^ Informatics & Data Science, Optum, Minnetonka, Minnesota.; ^3^ UnitedHealthcare Medicare & Retirement, Minneapolis, Minnesota.; ^4^ AARP Services, Inc., Washington, District of Columbia.

**Keywords:** older adults, resilience, social networks, pain outcomes

## Abstract

Depression, stress, and poor sleep have been associated with increased pain among older adults; positive resources, such as resilience and social networks, may help to buffer the impacts of these negative attributes on pain outcomes. The primary objective was to determine the relative effects of positive resources and negative attributes on pain outcomes among older adults with diagnosed back pain, osteoarthritis, and rheumatoid arthritis. The stratified study sample was identified from older adults ages ≥65 years. Members received a survey assessing positive resources (resilience, social networks), negative attributes (depression, stress, poor sleep), and pain outcomes (severity, interference). Opioid and other medication use was determined from pharmaceutical claims. After weighting to representative distributions of pain conditions and adjusting for survey response bias, multinomial logistic regressions were used to determine the relative associations of positive and negative attributes on pain outcomes. Among survey respondents (N = 4161), prevalence of self-reported pain severity and interference for no/mild, moderate, and severe categories was 61%, 21%, and 18%, and 67%, 16%, and 17%, respectively. In bivariate models, negative attributes of depression, stress, and poor sleep had stronger associations with pain severity and interference than the moderating effects of positive resources of high resilience and diverse social networks. In fully adjusted multivariate models, the strongest associations with moderate and severe pain severity and interference remained depression, stress, and poor sleep. Based on these results, multidimensional pain management strategies should include management of negative attributes along with enhancement of positive resources for effective management of chronic pain.

## Introduction

The negative attributes of depression,^1–7^ stress,^2,8–10^ and poor sleep^10–17^ have been associated with increased pain outcomes among older adults. There is general consensus that positive resources, such as resilience and social connectedness, can help to offset some of the impact of these negative attributes on health outcomes.^1,2,5,12,18–26^ Meanwhile, social science research has demonstrated consistent evidence of a negativity bias whereby negative stimuli, (eg, social strain, ambivalent ties, adverse events) influence physiological outcomes more strongly than do positive stimuli or events.^12,24,25^ It was of interest to understand if this phenomenon could be documented within the context of pain outcomes for the protective effects of positive resources relative to the detrimental impacts of negative attributes.

Among negative attributes, depression has been associated most consistently with pain severity and interference.^1–8,11,18^ Most consider the relationship to be bidirectional in that depression is associated with the likelihood of increased pain and pain is associated with the likelihood of increased depression.^3,6^ With the frequent co-occurrence of pain and depression, effective pain management strategies, of necessity, would imply the integration of mental health management. Although stress and pain are less often a focus of older adult research, stress overlaps with depression and shares a similar bidirectional association with pain outcomes.^2,8–10^ Perceived stress can have an independent impact on increasing pain severity and interference, or may augment depression and thus its association with pain.^2,8,9,21,24,26^

Improvements in sleep have been independently associated with improvements in pain.^13^ Although the relationship is considered bidirectional, positive sleep changes improved pain outcomes more strongly than reduced pain improved sleep outcomes.^13^ Poor sleep, although known to be associated with pain, is most often managed with pharmaceuticals and rarely treated as inherent to pain management or pain intervention strategies.^13,15^ Patients consider sleep improvement to be an important outcome of pain management; however, changes in sleep status, even when present, appear to be predominantly incidental to pain management strategies with little understanding of which factors may be driving the changes.^13,15^

Resilience is defined as a multidimensional construct synonymous with reduced vulnerability and with the ability to adapt to adversity with active coping abilities.^19^ Resilience has been shown to be protective in increasing the capacity to manage pain effectively, especially chronic pain.^2,5,19^ Resilience also appears to buffer depression and stress, which in turn indirectly reduces pain, rather than documented mechanisms for a direct pathway to pain per se.^5,18–21^ Notably, in one population-based study of older adults, widespread pain increased the likelihood of depressive symptoms by a factor of 4; meanwhile, resilience moderated the association by about 12%.^5^ Thus, although resilience buffered the pain–depression relationship significantly, negativity bias was evident in the stronger detrimental impact of the negative attribute.

Finally, social connectedness is generally measured as perceived social support evaluating the individual's perception of support, whether realized or not.^1,2,9,24,25^ More recently, a more quantitative approach to social networks has been designed as a “count” metric for various types of social experiences (eg, the number of contacts among family, friends, neighbors, religious communities, or clubs and organizations) with more diverse networks across types of social contacts conceived as being more stable and thus more protective.^23,26^ Regardless of the specific measure, however, perceived social support, social participation, or diverse social networks have demonstrated a protective effect on pain severity and subsequent functional abilities.^1,2,7,23–25^ Social support appears to buffer biological reactivity to stressful events and is health promoting either through better adherence to healthier behaviors and compliance with medical regimens or through minimizing psychological processes, such as depression.^1,23,24,26^

No published research studies to date were found that considered the relative impact of negative attributes and subsequent protective impact of positive resources on pain outcomes among older adults with Medicare Supplement plans (ie, Medigap).^27^ In the United States, government-funded Medicare covers adults ages 65 and older, as well as those younger than age 65 and disabled. Medicare fee-for-service plans (about 70% of all Medicare plans) pay about 80% of medical expenditures for these individuals but offer minimal prescription drug benefits. Those enrolled in these Medicare plans are personally responsible for obtaining additional insurance plans (ie, Medicare Supplement or Medigap plans) to cover the remaining 20% of medical expenses and more inclusive prescription drug coverage (Medicare Part D plans). Although most (about 90%) of those with original fee-for-service Medicare coverage have some type of supplemental insurance coverage, about 28% (currently about 11.8 million adults) have purchased Medigap coverage.^27^ Because this population may differ in demographic, socioeconomic, or health status characteristics from general older adult and/or specifically overall Medicare populations, it was of interest to determine the impact of negative attributes on pain severity and interference and, subsequently, the relative protective effects of positive resources on these same pain outcomes. This study adds to the pain literature by its focus on a relatively large population of older adults and its inclusion of multiple variables for both negative attributes and positive resources and their associations with pain outcomes, controlled for demographic, socioeconomic, health status, and pain-related medications.

Thus, the primary objectives were to (1) estimate the negative associations of depression, perceived stress, and poor sleep on pain severity and interference among older adults with documented pain conditions, and (2) estimate the relative protective effects of high resilience and more diverse social networks on these same pain outcomes. This research was covered under the New England Institutional Review Board #120160532.

## Methods

### Study sample

In 2016, approximately 5 million Medicare insureds were covered by an AARP^®^ Medicare Supplement plan insured by UnitedHealthcare Insurance Company and UnitedHealthcare Insurance Company of New York for New York certificate holders. These plans are offered in all 50 states, Washington DC, and various US territories. AARP Medicare Supplement insureds with AARP^®^ MedicareRx plans insured through UnitedHealthcare (about 55% of insureds) at least 65 years of age with a minimum of 12-month continuous medical and drug plan enrollment were used to generate a stratified sample mailing list for a pain-related survey. In addition, insureds must have had at least 1 of the following pain conditions identified from diagnosis codes: back pain (new or continuing), osteoarthritis, or rheumatoid arthritis. Those with cancer, trauma, or drug abuse were excluded. The stratified mailing list of 15,000 was drawn from a possible 327,685 insureds meeting eligibility criteria and included 7500 with back pain, 5000 with osteoarthritis, and 2500 with rheumatoid arthritis. Of survey respondents (4423; 29% response rate), those younger than age 65 years (N = 75) and those who did not answer the pain severity question (N = 187) were excluded. Thus, the final study population included 4161 survey respondents. Their responses were then weighted to adjust for nonresponse bias and to be representative of those with these pain conditions. This weighted study sample was used for the multivariate regression analyses.

### Pain-related survey

The mailed survey (54 questions) was developed by UnitedHealthcare to assess various aspects of pain including: negative attributes (depression, perceived stress, sleep quality), positive attributes (resilience, social networks), and pain outcomes of pain severity and pain interference. Other questions included self-reported use of physical therapy as a pain management strategy and body mass index (BMI) as a measure of obesity. The survey was mailed with a 2-month window to the stratified sample in May 2018 with a repeat mailing in June 2018 to those who had not yet responded.

### Pain outcomes

Pain severity and pain interference were assessed using the validated 3-item PEG assessment scale.^28^ The 3 items assessed pain severity (P), interference with enjoyment of life (E), and interference with general activity (G) on 0–10 scales. The 2 pain interference scales were averaged. Pain severity and pain interference were then categorized as no/mild (0–4), moderate (5–6), and severe (7–10). Because pain severity and pain interference were the intended outcome measures, those survey respondents missing pain severity or pain interference were excluded.

### Depression

Depression was measured using the self-reported Patient Health Quesionnaire-2 (PHQ-2).^29^ This 2-item depression screening tool is well validated and used frequently in clinical settings. The 4-level responses were scored 0 (*not at all*) to 3 (*nearly every day*) for a total score range of 0 to 6. The score was then dichotomized as 0–2 (not depressed) and 3–6 (depressed).

### Perceived stress

Perceived stress was measured using the Cohen perceived stress 4-item scale (PSS-4).^30^ Questions 1 and 4 were scored 0 (*never*) to 4 (*very often*) and questions 2 and 3 were reverse scored as 4 (*never*) to 0 (*very often*). To calculate a perceived stress score, respondents must have answered 2 of the 4 questions. Responses were averaged across the answered questions for a total score of 0 to 4. Average responses were then dichotomized as 0–2 (never to sometimes) as low stress and 3–4 (fairly often/very often) as high stress.

### Sleep quality: Pittsburgh Sleep Quality Index

Sleep quality was measured using the Pittsburgh Sleep Quality Index.^31^ For this study, the single-item sleep quality question (component 1) was used, with 4 responses from *very good* to *very bad*. The responses were dichotomized as good sleep (very good/fairly good) and poor sleep (fairly bad/very bad).

### Resilience

Resilience was measured using the 6-item Brief Resilience Scale.^32^ Responses, ranging 1 to 5, were scored if at least 3 of the 6 questions were completed and were averaged across the questions answered to give a range of scores from 1 to 5. Resilience was then dichotomized as follows: low (scores 1–3; responses *strongly disagree* to *neutral*); and high (scores 4–5; responses *agree* and *strongly agree*).

### Social network index

The Social Network Index counts the number of contacts across 4 different types of social connectedness: talking to friends, family or neighbors on the telephone per week; getting together with friends or relatives per week; attending church or religious services per month; attending meeting of clubs or organizations per month.^26^ Available responses included never, 1–2, 3–4, or 5+ times. Additionally, the index included being married (yes/no) scored 1/0. Respondents must have answered at least 3 of the possible 5 questions. Responses were scored 0 to 3 for the social connectedness items and 0 or 1 for the married question for a total score of 0 to 13. The Aung et al^23^ study was used as a guideline to categorize the social network index to 3 levels: limited (0–4), medium (5–7), and diverse (8–13).

### Pain-related medications

Opioids were identified from National Drug Codes (NDCs). Days of supply for the 1 year prior to the survey were calculated from prescriptions recorded in the pharmaceutical drug database. Based on the Healthcare Effectiveness Data and Information Set recommendation for use of opioids no longer than 14 days, opioid use was categorized as 0 to ≤14 days (low risk) and ≥15 days (high risk).^33^

Opioids also were categorized into 6 mutually exclusive categories based on US Drug Enforcement Administration opioid drug schedules for acceptability of medical use and potential for abuse or dependency. Those categories are: (1) long acting; (2) short acting, other Schedule II; (3) short acting, oxycodone; (4) short acting, hydrocodone; (5) short acting, Schedule III-IV and nalbuphine; and (6) tramadol.^34^

Other medications often used concurrently with opioids to manage pain included other pain medications (nonsteroidal anti-inflammatory drugs [NSAIDs] and muscle relaxants) and mental health medications (benzodiazepines). Benzodiazepines, muscle relaxants, and NSAIDs were defined from NDCs included in the drug classes for general use benzodiazepines, muscle relaxants, or NSAIDs.

### Covariates

Covariates were included to characterize categories of pain severity and pain interference and to adjust for other risk factors. These covariates included measures of demographics, socioeconomic factors, health status, and other characteristics taken from health plan eligibility and administrative medical claims.

Demographic questions included age and sex. Age groups were defined as: 65–69; 70–74; 75–79; 80–84; and ≥85 years. Geographical location (Northeast, South, Midwest or West); metropolitan area (urban or other); and low (less than 15% nonwhite), medium (15% to 59% nonwhite), and high (≥60% nonwhite) minority areas were geocoded from zip codes as determined by the US Census Bureau. AARP Medicare Supplement plan types were grouped by cost-sharing levels, including high-level coverage plans with minimal co-payments or deductibles, medium-level coverage plans with relatively more co-payments or deductibles, and all other plans. A measure of health services access was calculated as primary care physicians (PCPs) per 100,000 capita. Level of medical services utilization from medical claims was calculated as the Hierarchical Condition Category (HCC) score.^35^ This score is used by the Centers for Medicare & Medicaid Services to risk adjust medical payments across various medical plans according to the health status of the different insured populations. HCC subgroups were defined as follows and utilized to control for health status: HCC scores <0.5, HCC scores 0.5 to <1.2, HCC scores 1.2 to <2.8, and HCC scores ≥2.8. Physical therapy sessions were identified from self-reported utilization as a strategy used for pain management. BMI was calculated from self-reported weight and height: weight in kilograms/height in meters squared. Obesity was defined as BMI ≥30.

### Statistics

#### Weighting to adjust for survey nonresponse bias and stratified sampling

Propensity weighting was used to adjust for potential selection bias often associated with survey response to enhance the generalizability of these findings. The propensity weighting utilized available information about the aforementioned demographic, socioeconomic, and health status variables that could potentially influence survey response. The utility of such propensity weighting models to adjust for external validity threats is described elsewhere.^36,37^ In addition, survey responses were weighted to achieve national representation of the AARP Medicare Supplement population with defined pain conditions of back pain, osteoarthritis, and rheumatoid arthritis and 1 year of medical and drug plan eligibility.

#### Demographics and logistic regression models

Demographic variables were unilaterally tested across pain severity and pain interference categories using chi-square or *t* tests for categorical or continuous variables, respectively. Characteristics associated with pain categories comparing moderate or severe pain severity or pain interference to no/mild pain severity or pain interference, respectively, were determined using multinomial logistic regression models. Because depression and high stress were strongly correlated (>0.60), separate models were developed for depression and high stress. The study team initially established a base model that included demographic (age, sex, minority status, location, region), socioeconomic (plan type), access to care (PCPs per 100,000), and health status (HCC score categories, obesity, pain conditions of back pain, osteoarthritis, and rheumatoid arthritis) variables listed in Tables 1 and 2. The team subsequently added each of the psychosocial, medication, and physical therapy variables listed in Tables 3 and 4 one at a time (adjusted bivariate odds ratios [ORs]) and then all variables in full models for depression and stress separately (full model ORs). All analyses were completed using SAS Enterprise Guide Version 7.1 (SAS Institute Inc., Cary, NC, USA).

**Table 1.** Unadjusted Demographic Characteristics by Pain Severity Levels

		*Pain severity*			
	*All mean or %*	*None/mild (0–4) mean or %*	*Moderate (5–6) mean or %*	*Severe (7–10) mean or %*	P *value*
Number	4161	2447	910	804	
Sex					
Female	67.2	63.0	73.5	72.8	<0.0001
Male	32.8	37.0	26.5	27.2	
Age groups	75.9	75.6	76.5	75.9	0.004
65–69	21.3	22.0	19.2	21.6	0.04
70–74	25.7	26.8	23.6	25.0	
75–79	24.7	24.9	24.1	24.8	
80–84	14.0	13.1	16.4	14.1	
≥85	14.2	13.2	16.7	14.6	
Minority (from zip codes)					
Low	50.8	52.3	48.2	49.3	0.04
Median	45.1	43.4	48.0	47.0	
High	2.9	2.9	2.5	3.4	
Location					
Metro	83.4	83.6	85.2	80.7	0.04
Other	16.6	16.4	14.8	19.3	
Region					
Midwest	24.8	25.8	23.6	22.8	0.17
Northeast	19.7	20.0	20.1	18.4	
South	26.7	25.2	27.5	30.5	
West	28.7	28.8	28.7	28.4	
Access to health care					
PCPs per 100,000	133.7	135.0	131.6	132.0	0.08
Plan type coverage					
High	77.2	77.3	76.7	77.7	0.88
Medium	2.6	2.6	2.4	3.0	
Other	20.1	20.2	20.9	19.3	
HCC Score					
<0.50	20.5	25.4	15.2	11.8	<0.0001
0.50 to <1.20	45.7	47.6	44.2	41.8	
1.20 to <2.80	28.7	23.4	35.1	37.9	
≥2.8	5.0	3.6	5.6	8.5	
PHQ-2 (Depression)					
0–2	85.5	94.1	81.1	64.4	
≥3	14.2	5.8	18.7	35.1	
Perceived stress					
Low (0–1)	57.9	69.8	46.5	34.7	<0.0001
Moderate (2)	33.4	26.6	42.0	44.3	
High (3–4)	8.1	2.9	10.7	20.7	
Resilience scale					
Low (<4)	57.4	47.0	67.3	78.2	<0.0001
High (≥4)	42.0	52.5	32.1	21.4	
Social Network Index					
Limited (0–4)	27.1	22.2	30.6	38.1	<0.0001
Medium (5–7)	42.0	42.3	41.4	41.5	
Diverse (≥8)	26.6	31.2	22.6	16.9	
Pittsburgh Sleep Quality Index					
Good	77.2	86.0	70.4	58.3	<0.0001
Poor	21.8	13.3	28.4	40.4	
Opioid days of supply					
None or used ≤14 days	66.2	77.5	58.9	39.8	<0.0001
≥15 days	33.8	22.5	41.1	60.2	
Opioid category (initial)					
1: Long acting	3.6	1.9	3.6	8.8	<0.0001
2: Short acting, other Schedule II	2.0	1.3	2.4	3.7	<0.0001
3: Short acting, oxycodone	14.2	11.0	14.1	24.3	<0.0001
4: Short acting, hydrocodone	25.4	20.8	27.8	36.4	<0.0001
5: Short acting, Schedule III - IV	5.1	4.4	5.7	6.7	0.03
6: Tramadol	20.5	16.6	25.5	26.9	<0.0001
Medications					
NSAIDs Rx	31.2	28.2	34.7	36.6	<0.0001
Benzodiazepine Rx	19.3	15.2	21.4	29.4	<0.0001
Muscle relaxant Rx	13.8	11.5	14.8	19.7	<0.0001
Pain conditions (from diagnosis codes)					
Low back pain	56.1	54.8	56.7	59.3	0.07
Osteoarthritis	56.1	53.7	58.6	60.7	0.0006
Rheumatoid arthritis	18.9	17.3	20.6	21.9	0.005
BMI					
Obese (≥ 30)	30.7	26.1	35.1	39.7	<0.0001
Not obese	65.9	70.5	61.8	56.7	
Physical therapy (self-reported)	33.8	31.5	37.6	36.7	<0.0001

BMI, body mass index; HCC, Hierarchical Condition Category; NSAIDs, nonsteroidal anti-inflammatory drugs; PHQ-2, Patient Health Questionnaire-2; PCP, primary care physician; Rx, prescription.

**Table 2.** Unadjusted Demographic Characteristics by Pain Interference Levels

		*Pain interference*			
	*All*	*None/mild (0–4)*	*Moderate (5–6)*	*Severe (7–10)*	P *value*
		*mean or %*	*mean or %*	*mean or %*	
Number	4146	2663	700	783	
Sex					
Female	67.1	64.7	69.3	73.6	<0.0001
Male	32.9	35.3	30.7	26.4	
Age groups	75.9	75.8	76.3	75.8	0.24
65–69	21.3	21.9	18.1	22.4	0.54
70–74	25.8	26.0	26.0	25.0	
75–79	24.7	24.8	25.6	23.8	
80–84	14.0	13.6	14.6	14.8	
≥85	14.2	13.9	15.7	14.1	
Minority (from zip codes)					
Low	50.8	52.0	46.3	51.1	0.04
Median	45.1	43.7	49.4	45.9	
High	2.9	2.9	3.1	2.7	
Location					
Metro	83.4	83.7	85.1	80.6	0.04
Other	16.6	16.3	14.9	19.4	
Region					
Midwest	24.8	25.9	23.1	22.4	0.005
Northeast	19.7	20.7	19.4	16.7	
South	26.7	25.0	28.0	31.6	
West	28.7	28.2	29.4	29.4	
Access to health care					
PCPs per 100,000	133.7	134.2	133.1	132.3	0.53
Plan type coverage					
High	77.2	76.4	77.6	79.7	0.12
Medium	2.6	2.5	3.6	2.3	
Other	20.1	21.1	18.9	18.0	
HCC Score					
<0.50	20.6	25.1	13.3	11.9	<0.0001
0.50 to <1.20	45.7	47.4	43.6	41.6	
1.20 to <2.80	28.7	24.0	37.1	37.3	
≥2.8	5.0	3.5	6.0	9.2	
PHQ-2 (Depression)					
0–2	85.5	94.9	79.4	59.4	<0.0001
≥3	14.3	5.0	20.4	40.2	
Perceived stress					
Low (0–1)	58.0	70.2	41.9	30.8	<0.0001
Moderate (2)	33.4	26.5	45.4	46.0	
High (3–4)	8.1	2.7	12.0	22.9	
Resilience scale					
Low (<4)	57.5	47.4	72.4	78.3	<0.0001
High (≥4)	42.0	52.1	27.3	21.2	
Social Network Index					
Limited (0–4)	27.0	21.2	32.4	42.0	<0.0001
Medium (5–7)	42.0	43.0	41.0	39.7	
Diverse (≥8)	26.6	31.3	22.0	14.7	
Pittsburgh Sleep Quality Index					
Good	77.3	85.8	69.0	56.1	<0.0001
Poor	21.8	13.5	30.0	42.8	
Opioid days of supply					
None or used ≤14 days	66.2	76.4	58.6	38.3	<0.0001
≥15 days	33.8	23.6	41.4	61.7	
Opioid category (initial)					
1: Long acting	3.6	1.9	3.9	9.3	<0.0001
2: Short acting, other Schedule II	2.0	1.5	2.0	3.6	0.002
3: Short acting, oxycodone	14.2	10.6	16.7	24.0	<0.0001
4: Short acting, hydrocodone	25.3	21.0	28.3	37.4	<0.0001
5: Short acting, Schedule III - IV	5.2	4.5	5.3	7.2	0.01
6: Tramadol	20.5	17.3	25.4	27.0	<0.0001
Medications					
NSAID Rx	31.2	29.0	35.1	35.5	0.0001
Benzodiazepine Rx	19.3	15.9	21.7	28.7	<0.0001
Muscle relaxant Rx	13.8	11.3	17.0	19.3	<0.0001
Pain conditions (from diagnosis codes)					
Low back pain	56.1	54.3	57.4	60.8	0.004
Osteoarthritis	56.2	54.2	59.0	60.4	0.002
Rheumatoid arthritis	18.8	17.6	20.9	20.8	0.04
BMI					
Obese (≥30)	30.7	26.1	38.3	39.5	<0.0001
Not obese	66.0	70.6	58.4	57.1	
Physical therapy (self-reported)	33.8	30.7	40.0	39.0	<0.0001

BMI, body mass index; HCC, Hierarchical Condition Category; NSAIDs, nonsteroidal anti-inflammatory drugs; PHQ-2, Patient Health Questionnaire-2; PCP, primary care physician; Rx, prescription.

**Table 3.** Regression Adjusted Odds Ratios for Positive Resources and Negative Attributes Associated with Pain Severity Levels

	Adjusted^*^ bivariate odds ratios	Full model^*^ depression odds ratios	Full model^*^ stress odds ratios
Pain level: Moderate vs Mild/None (N = 64,904 weighted moderate pain severity)			
Depression	4.1	2.6	–
Stress–high	5.0	–	3.2
Poor sleep quality	2.8	2.3	2.3
High resilience	0.5	0.6	0.6
Medium social network index	0.7	0.9	0.9
Diverse social network index	0.5	0.7	0.7
Physical therapy	1.1	1.1	1.1
Opioid use ≥15 days	2.2	1.9	1.9
NSAID Rx	1.5	1.4	1.4
Benzodiazepine Rx	1.5	1.1	1.1
Muscle relaxant Rx	1.4	1.0^**^	1.0^**^
Pain level: Severe vs Mild/None (N = 54,723 weighted severe pain severity)			
Depression	9.4	4.7	–
Stress-high	9.2	–	4.7
Poor sleep quality	4.7	3.1	3.3
High resilience	0.3	0.5	0.4
Medium social network index	0.5	0.8	0.7
Diverse social network index	0.3	0.6	0.5
Physical therapy	1.2	1.2	1.1
Opioid use ≥15 days	4.6	3.5	3.6
NSAID Rx	1.4	1.2	1.1
Benzodiazepine Rx	2.3	1.4	1.5
Muscle relaxant Rx	2.4	1.4	1.4

^*^ Adjusted for age, sex, minority, region, location, plan type, PCP access, HCC score, obesity, pain conditions (back pain, osteoarthritis, and rheumatoid arthritis).

^**^ Not significant. All other variables significant *P* < 0.0001.

HCC, Hierarchical Condition Category; NSAIDs, nonsteroidal anti-inflammatory drugs; PCP, primary care physician; Rx, prescription.

**Table 4.** Regression Adjusted Odds Ratios for Positive Resources and Negative Attributes Associated with Pain Interference Levels

	Adjusted^*^ bivariate odds ratios	Full model^*^ depression odds ratios	Full model^*^ stress odds ratios
Pain interference: Moderate vs None/Mild (N = 49,644 weighted moderate pain interference)			
Depression	4.5	2.7	–
Stress–high	5.4	–	3.2
Bad sleep quality	3.0	2.4	2.5
High resilience	0.4	0.5	0.5
Medium social network index	0.6	0.7	0.7
Diverse social network index	0.4	0.5	0.5
Physical therapy	1.4	1.4	1.4
Opioid use ≥15 days	2.1	1.7	1.7
NSAID Rx	1.5	1.4	1.4
Benzodiazepine Rx	1.5	1.1	1.2
Muscle relaxant Rx	1.8	1.3	1.3
Pain interference: High vs None/Mild (N = 52,935 weighted severe pain interference)			
Depression	13.3	7.0	–
Stress–high	12.2	–	6.3
Bad sleep quality	5.3	3.5	3.7
High resilience	0.3	0.5	0.4
Medium social network index	0.4	0.6	0.5
Diverse social network index	0.2	0.4	0.4
Physical therapy	1.4	1.4	1.3
Opioid use ≥15 days	4.6	3.4	3.6
NSAID Rx	1.4	1.2	1.2
Benzodiazepine Rx	2.2	1.2	1.3
Muscle relaxant Rx	2.5	1.5	1.5

^*^ Adjusted for age, sex, minority, region, location, plan type, PCP access, HCC score, obesity, pain conditions (back pain, osteoarthritis, and rheumatoid arthritis). All variables significant *P* < 0.0001.

HCC, Hierarchical Condition Category; NSAIDs, nonsteroidal anti-inflammatory drugs; PCP, primary care physician; Rx, prescription.

## Results

Overall, 4423 AARP Medicare Supplement insureds responded to the survey (29% response rate). Of these, 94% (N = 4161) met eligibility criteria and were included in the study. Responses were subsequently weighted to a nationally representative population of 308,443 insureds with pain conditions. The distribution of pain conditions among survey respondents were 56%, 56%, and 19% for back pain, osteoarthritis and rheumatoid arthritis, respectively; the weighted distributions were 29%, 77%, and 8%, respectively. Despite the differences in pain condition ratios, the weighting resulted in minimal differences in the prevalence of the pain severity or pain interference categories or of any of the variables listed in Tables 1 and 2; consequently, only the survey responses are shown. Survey respondents for pain severity (N = 4161) and pain interference (N = 4146) were 84% correlated; hence, demographics for the 2 outcomes were similar. Survey respondents for severity and interference outcomes were mostly female, 70–74 years of age, and white (Tables 1 and 2). The prevalence of HCC health status groups (HCC scores <0.5, HCC scores 0.5 to <1.2, HCC scores 1.2 to <2.8, and HCC scores ≥2.8) were as follows: 21%, 46%, 29% and 5% for both pain severity and interference.

Among survey respondents, the prevalence of no/mild, moderate, and severe pain severity levels were 59%, 22% and 19%, respectively; and 64%, 17% and 19%, respectively, for pain interference (Tables 1 and 2). The prevalence of depression, high stress, and poor sleep were 14%, 8% and 22%, respectively, for both pain severity and interference. The prevalence of high resilience and diverse social networks were likewise similar for both pain severity and interference. About one third used opioids 15 days or longer; the most common opioids were hydrocodone and tramadol.

### Characteristics associated with pain severity levels: moderate and severe vs. no/mild

Although attenuated by 37% to 50% with the addition to the models of positive resources and pain-related medications, depression and high stress maintained the strongest associations with both moderate and severe pain severity (ORs 2.6–3.2 moderate; 4.7 severe) (Table 3). Poor sleep, attenuated by 20% to 35%, had the second highest impact. High resilience attenuated moderate and severe pain severity by 40%–60%; diverse social networks by 30%–40%; and medium social networks by 10%–30%. Opioid use was attenuated by 15% to 25%; benzodiazepine use by 25%–40%. High resilience was relatively more protective for pain severity than social networks, medium or diverse.

### Characteristics associated with pain interference levels: moderate and severe vs. no/mild

As expected, negative attributes and positive resources had similar associations with pain interference as with pain severity. The impact of depression and stress on pain interference was attenuated by 40%–50% with the addition to the models of positive resources and pain-related medications. Nevertheless, depression and high stress maintained the strongest associations with both moderate and severe pain interference (ORs 2.7–3.2 moderate; 6.3–7.0 severe) (Table 4). Poor sleep, attenuated by 20%–30%, had the second highest impact. High resilience attenuated moderate and severe pain interference by 50%–60%; diverse social networks by 50%–60%; and medium social networks by 30%–50%. Opioid use was attenuated by 20%–25%; benzodiazepine use by 25%–45%. Positive resources, especially medium and diverse social networks, were relatively more protective for pain interference than for pain severity.

## Discussion

In this population of AARP Medicare Supplement insureds, the weighted prevalence of no/mild, moderate, and severe pain severity and pain interference were 61%, 21%, and 18% and 67%, 16%, and 17%, respectively. Although measurement scales and definitions of “moderate and severe” pain severity differ somewhat in the literature, the prevalence of pain severity in this population with pain conditions is in general agreement with other studies focused on study populations with chronic pain issues (62%–66% no/mild pain; 34%–38% moderate/severe pain).^6,8,22^

The characteristics most strongly associated with moderate and severe pain severity and interference were depression and stress.^2,3,5^ Although attenuated by positive resources and pain-related medications, ORs associated with moderate and severe pain severity and interference for depression and stress ranged from 3.0–5.0 for pain severity and 3.0–7.0 for pain interference. Of note, stress demonstrated a stronger association with moderate pain than depression. The overall strong associations of depression and stress are consistent with other studies, although the magnitude of the associations were difficult to compare because of differences in study populations, measurement tools for the various scales, and analytic approaches with most studies utilizing total scores as continuous variables in linear regression models.^1–7,11^ The magnitude of the impact for high stress was comparable to that of depression and is especially noteworthy because stress is less often studied in older adult populations.

Sleep was the second strongest characteristic associated with moderate and severe pain severity and interference.^10–15,17^ Despite attenuation by positive resources and pain-related medications, ORs ranged from 2.0–3.0 for moderate and severe pain severity and 3.0–4.0 for moderate and severe pain interference. In longitudinal studies, improvements in sleep have predicted improvements in pain severity.^13^ However, despite an importance attached to good sleep by patients, sleep is generally treated as an incidental symptom associated with pain and managed with pharmaceuticals.^13^ Poor sleep has been associated with depression as well as with pain; however, management of sleep problems has not been extensively integrated into either pain or depression management interventions.^13,15^

High resilience, medium, and diverse social networks reduced the likelihood of moderate and severe pain severity. Additionally, in fully adjusted models controlling for positive resources and pain-related medications, depression and stress were attenuated by about 50% and poor sleep by about 30%. The study team could find no other studies that compared the magnitude of attenuation for both resilience and/or social networks on pain severity. Resilience had been shown to attenuate the depression and pain relationship by about 12%, which may indicate a stronger buffering impact in this study population.^5,18^ Social support, although often using different metrics, has been shown to demonstrate a protective effect on depression and stress along with pain severity, consistent with these results.^1,2,7,22–26^ Most research studies, however, use continuous variables for relevant scales in linear regression models so direct comparisons of magnitudes of impact are difficult.^1–4,6,10–13,19–23^ Nevertheless, a consistent negativity bias has been demonstrated previously for social support and health outcomes, with social strain being more powerful than supportive social support on cardiovascular risk factors^24^ and ambivalent relationships outweighing supportive relationships for sleep outcomes.^25^ Thus, although present study results cannot be compared to other studies directly, the negativity bias is clearly evident in the data in the magnitude of the impacts for depression, stress, and poor sleep despite positive resources and pain-related medications.

Likewise, the strongest characteristics associated with moderate and severe pain interference, while attenuated, remained depression, stress, and poor sleep.^2,4,8^ High resilience, medium, and diverse social networks significantly reduced the likelihood of moderate and severe pain interference.^2,19,22^ In fully adjusted models, depression and stress were attenuated by about 40%–50% and poor sleep by about 20%–30%. In other studies, social support has been associated with better physical quality of life scores, and improved functional abilities^1,2,16,23^; meanwhile, resilience has been associated consistently with reduced depression.^2–4,6,7,11^ Nevertheless, the negative attributes remained more strongly associated with pain interference despite positive resources or pain-related medications.^5,12,18,24,25^

Few research studies focused on survey outcomes for positive resources, negative attributes and pain outcomes have had access to pain-related medication data, either self-reported or from database records.^4,5,19,22^ Thus, the present study is relatively unique in that the study team was able to incorporate the documented use of opioids, benzodiazepines, NSAIDs, and muscle relaxants. Although about 34% used opioids 15 days or longer, the most common use of hydrocodone and tramadol would indicate the management of low-level chronic pain. As expected, use of pain-related medications was most highly associated with severe pain. Nevertheless, the results would indicate that, despite controlling for medication use and positive resources, these patients continued to self-report both moderate and severe levels of pain severity and interference with associated mental health issues.

First-line treatment protocols for pain management have relied largely on opioids and other pharmaceutical analgesics.^33^ These results would indicate that multidimensional interventions concurrently addressing negative attributes–especially depression, stress, and sleep issues–as well as promoting positive resources also may be required for effective pain management. Non-pharmacological approaches targeting mental health and sleep issues that have demonstrated success among older adults might include cognitive behavioral therapy for pain and/or insomnia, mindfulness for stress and pain, or optimized antidepressant therapy with pain management.^13,15,38,39^ Unfortunately, these programs require additional time and resources not generally available to many physicians. Furthermore, published pain interventions often are based on research studies that involve small study populations and lack generalizability to older adults. Physical therapy, exercise therapy, yoga, and progressive muscle relaxation along with psychological interventions have been recommended, especially for back pain issues, but have not been utilized consistently.^7,40^ In addition, research is needed that considers pain management within a holistic context including not only mental and physical health but also the broader social determinants of health (eg, transportation, access to healthy food, living arrangements). Thus, although non-pharmacological approaches to pain management do exist, limitations of physician time, available program resources, and recommended interventions with documented results have made practical applications difficult. Future research might include alternative models of health care delivery with more inclusive services, such as Medicare Advantage or other integrated care delivery models.

This study has some limitations. The study population of AARP Medicare Supplement insureds may not generalize to all older adults or other Medicare or Medicare Supplement insureds. Pharmacy databases confirmed prescription purchases but the study team had no indication of whether patients actually consumed the drugs as directed. Depression, stress, sleep, resilience, social networks, and pain outcomes were self-reported and may be subject to bias. Strengths of the study include a relatively large survey population with information on positive resources, negative attributes and pain-related medications that could be directly compared in multivariate regression models.

## Conclusions

Overall, in this population of Medicare Supplement insureds, about 40% reported either moderate or severe pain severity or interference. Although resilience and social networks attenuated the negative impacts of depression, stress, and poor sleep, the strongest characteristics associated with moderate and severe pain severity and interference remained the negative attributes. Despite positive resources and controlling for pain-related medications, mental health issues were most strongly associated with increased pain outcomes. Thus, based on these results, multidimensional pain management strategies should include management of depression, stress, and poor sleep along with promotion of positive resources, such as resilience or social connectedness, for effective management of chronic pain.
